# Ti_3_AlC_2_/Pd Composites for Efficient Hydrogen Production from Alkaline Formaldehyde Solutions

**DOI:** 10.3390/nano12050843

**Published:** 2022-03-02

**Authors:** Xiaogang Liu, Wenjie Chen, Xin Zhang

**Affiliations:** College of Chemistry and Chemical Engineering, Xinyang Normal University, Xinyang 464000, China; chen2wen9jie5@163.com (W.C.); zhangxin109zz@163.com (X.Z.)

**Keywords:** Ti_3_AlC_2_ MAX phase, Pd nanoparticles, hydrogen production, formaldehyde

## Abstract

Research on catalytic oxidation in a promising but mild manner to remove formaldehyde and produce hydrogen is rarely reported. Here, the use of the Ti_3_AlC_2_ MAX phase as support for palladium nanoparticles was explored for the hydrogen generation from alkaline formaldehyde solution at room temperature. The results showed that Ti_3_AlC_2_/Pd catalyst with 3 wt% Pd loading had a much higher capability for hydrogen production than conventional Pd nanoparticles. In addition, by further optimizing the formaldehyde concentration, NaOH concentration, and the reaction temperature, the hydrogen production rate could be further increased to 291.6 mL min^−1^g^−1^. Moreover, the obtained apparent activation energy of the Ti_3_AlC_2_/Pd catalyzed hydrogen production reaction is 39.48 kJ mol^−1^, which is much lower than that of the literature results (65 kJ mol^−1^). The prepared Ti_3_AlC_2_/Pd catalysts as well as the catalytic process could act as a “two birds with one stone” effect, that is, they not only eliminate noxious formaldehyde but also generate clean hydrogen.

## 1. Introduction

With the development of science and technology, the demand for energy in modern society is increasing rapidly. However, the stock of coal, oil, and other resources on the earth we live on is limited. The development and utilization of a large amount of fossil energy has not only greatly reduced the inventory of non-renewable energy but has also worsened environmental problems and endangered the physical and mental health of human beings. Therefore, it is necessary to transform energy consumption patterns and develop clean energy. Developing green energy including solar energy [1,2], wind energy [3,4], water energy [5], and hydrogen energy [6,7] is a reliable and alternative choice for dealing with the global energy crisis and environmental pollution. Hydrogen energy, which has a wide range of sources and applications, has a broader application prospect. Hydrogen energy is high-efficiency clean energy with high energy density, renewable energy, convenient storage and transportation, and no carbon dioxide emissions [8,9]. At present, the most widely used hydrogen production method is fossil fuels, such as coal, oil, and other hydrocarbon reforming hydrogen production [10,11,12,13]. However, hydrogen produced by these methods generally leads to the emission of carbon oxides, which is not environmentally friendly. In addition, there are biological [14] and electrocatalytic [15] methods for hydrogen production. However, the immature development of biological hydrogen production technology is greatly limited by its high cost and low hydrogen production efficiency, which makes it impractical to produce hydrogen on a large scale. Therefore, it is necessary to develop highly efficient and stable hydrogen production procedures and highly active catalysts.

Indoor air quality is currently a concern for human society. Formaldehyde, a universal but important air pollutant, exists in various indoor environments, including houses, offices, and industries. The emission sources of formaldehyde are diverse, and long-term exposure can cause serious health problems. Therefore, this molecule has become a primary pollutant that needs to be eliminated first. Some regulations are creating new challenges, and various effective removal techniques have been developed so far. Adsorption of formaldehyde on activated carbon [16,17] or other adsorbents such as zeolite materials [18,19] with large surface areas and multistage pore structures is also proposed as an efficient way of eliminating formaldehyde. However, formaldehyde cannot be permanently eliminated and must undergo subsequent secondary degradation. Complete catalytic oxidation of formaldehyde is an attractive alternative to other proposed procedures. However, one of the products of complete oxidation of formaldehyde is CO_2_, which is generally deemed as the chief culprit of global warming. Therefore, the development of new catalysts exhibiting high formaldehyde-oxidation catalytic activity and preferably free of CO_2_ generation is highly appreciated.

**Figure 1:**
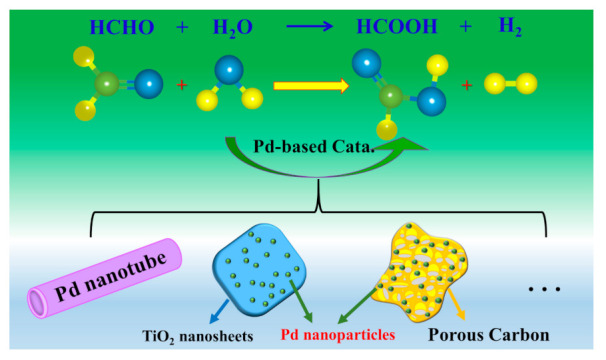
Schematic diagram of Pd-based catalysts’ structure designed to catalyze hydrogen production from formaldehyde.

Earlier research on the Cannizzaro reaction indicated that formaldehyde was very likely to be an intermediate for the production of H_2_ during the storage of alkaline nuclear waste [20]. The experimental results suggested that one of the hydrogen atoms in hydrogen originated from formaldehyde and the other from water molecules. This typical process not only makes it possible to eliminate formaldehyde but also provides an alternative but effective way to produce hydrogen. However, this process requires a high concentration of alkaline medium (usually 18 M), and suffers from low H_2_ production efficiency [21]. Thereby, the harsh reaction conditions and low hydrogen production make its application impractical. A follow-up study by Hu et al. [22] in 2014 showed that Pd nanotubes could serve as a highly efficient catalyst to inhibit the Cannizzaro reaction and facilitate hydrogen production from basic formaldehyde aqueous solution under room temperature. After optimizing the reaction conditions, the hydrogen production over as-prepared Pd nanotube could reach 170 mL g^−1^min^−1^ even under a lower alkaline concentration of 0.1 M. However, the high and fluctuating costs and the limited resources of noble metals limit their expensive use. Although pure Pd nanotubes [22], Pd supported on TiO_2_ (001) nanosheets [23], 3D porous carbon [24], and other supports [25] have been proven effective for the oxidation of formaldehyde at low temperatures (as illustrated in Figure 1), they usually require a large amount of noble metal loading, which greatly limits their further widespread applications. Therefore, the decrease of the noble-metals loading in an active component while maintaining their satisfactory catalytic activity is a key scientific issue.

The MAX phases are ternary transition metal carbide and/or nitride with a layered hexagonal crystal structure [26,27]. The unique structure endows them the dual properties of both metals and ceramics, that is, they are machinable and possess high electronic conductivities, are lightweight, and oxidation resistant [28,29]. Further studies by Ng et al. showed the potential of Ti_3_AlC_2_ as a catalyst in selective oxidation reactions, which was most likely derived from the non-stoichiometric oxide surface layer containing oxygen vacancies [30].

To the best of our knowledge, no hydrogen production reactions from formaldehyde have been reported so far where Ti_3_AlC_2_ MAX phase was used as either a catalyst or support. More importantly, the fundamental chemical principles responsible for the supported Pd nanoparticle-mediated hydrogen production from a basic HCHO aqueous solution remain unclear. Herein, the use of Ti_3_AlC_2_ MAX phase as support for palladium nanoparticles was explored for the hydrogen generation from alkaline formaldehyde solution at room temperature. More specifically, the rates of hydrogen generation over Ti_3_AlC_2_/Pd with Pd loading of 3 wt% could be increased up to 291.6 mL min^−1^g^−1^, far exceeding that of its Pd nanoparticle counterpart. In addition, experimental parameters such as reaction temperature, NaOH concentrations, and formaldehyde concentration were systematically investigated to determine the effect on the hydrogen production rates. The prepared Ti_3_AlC_2_/Pd catalysts, as well as the catalytic formaldehyde oxidation process could act as a “two birds with one stone” effect, that is, they not only eliminate noxious formaldehyde but also generate clean hydrogen.

## 2. Materials and Methods

### 2.1. Materials

The Ti_3_AlC_2_ (400 meshes) powder was provided by Beijing Kaifa Tetao Technology Co., Ltd. (Beijing, China) Palladium (II) Chloride (PdCl_2_, Adamas, RG, Shanghai, China), sodium hydroxide (NaOH, Greagent, AR, Shanghai, China), formaldehyde (HCHO, Adamas, 36–38% in water, ACS), and sodium borohydride (NaBH_4_, Greagent, AR) were used without any further purification.

### 2.2. Synthesis of Ti_3_AlC_2_/Pd Catalysts

The Ti_3_AlC_2_/Pd catalysts were prepared via an impregnation-reduction method. Specifically, 0.2 g of Ti_3_AlC_2_ powder (400 meshes, Beijing Kaifa Tetao Technology Co., Ltd., Beijing, China) was added to 50 mL of deionized water and ultrasonicated for 30 min. Then, an appropriate amount of H_2_PdCl_4_ solution and 20 mL of 0.5 M NaBH_4_ (freshly prepared) aqueous solution were added in sequence into the above solution. The mixed solution was continuously stirred for 24 h to complete the reduction reaction. After that, the suspension was centrifuged and washed thoroughly with ethanol and deionized water four times and then dried in an oven at 60 °C for 12 h. Subsequently, the sample was transferred to a crucible and calcined at 450 °C in an argon atmosphere for 2 h. The prepared samples were denoted as Ti_3_AlC_2_/Pd-1%, Ti_3_AlC_2_/Pd-3%, and Ti_3_AlC_2_/Pd-5%, according to the theoretically molar content of palladium, respectively. For comparison, Ti_3_AlC_2_/Pd catalysts with a Pd loading mass ratio of 3 wt% prepared without calcination at 450 °C in an argon atmosphere were denoted as Ti_3_AlC_2_/Pd-3%-uc.

### 2.3. Material Characterizations

Scanning electron microscope (SEM) images were taken using a field-emission scanning electron microscope (Hitachi, S 4800, Tokyo, Japan) operated at an accelerating voltage of 5 kV. Transmission electron microscopy (TEM) images were obtained on a transmission electron microscope (FEI, Tecnai G2 F 20, Pittsburgh, PA, USA) at an acceleration voltage of 200 kV. The Benchtop X-ray diffractometer (Rigaku Mini Flex 600, Austin, TX, USA), operating at 40 kV and 15 mA with Cu Kα radiation, was used to collect the X-ray powder diffraction (XRD) patterns. X-ray photoelectron spectrometry (XPS) was carried out on the K-ALPHA 0.5 EV X-ray photoelectron spectrometer using an Al Kα source. The infrared spectra were obtained on a Fourier Transform Infrared (FTIR) spectroscopy (iS50, Nicolet, Green Bay, WI, USA). The contents of Pd were measured by inductively coupled plasma mass spectrometry (ICP-MS, Agilent 8900, Santa Clara, CA, USA).

### 2.4. Activity Tests

As for the catalytic process, sodium hydroxide aqueous solution with appropriate concentrations was added into the glass bottle, and then 15 mg of catalyst was added. The mixed solution was sonicated for 5 min to make the catalyst uniformly dispersed, and then a certain volume of formaldehyde (37 wt%) was added. The bottle was sealed, and the reaction started under continuous stirring. The hydrogen production reaction was started while the solution was stirred vigorously. Only H_2_ and no other gas species were analyzed by a GC (gas chromatograph) equipped with a TCD detector and argon as carrier gas. During the reaction process, a certain volume of evolved gas was derived and injected into the chromatography inlet to analyze the generated hydrogen. Each experiment was repeated three times to ensure the accuracy of hydrogen production.

## 3. Results

**Figure 2:**
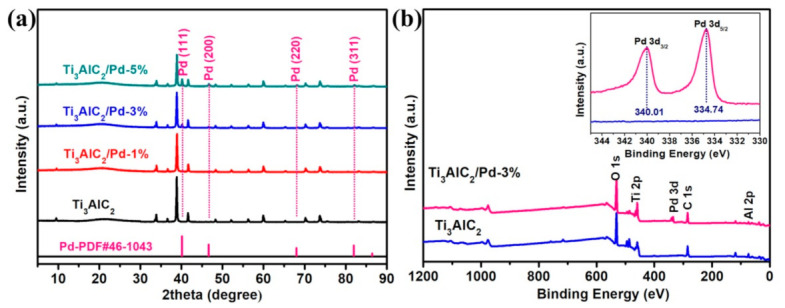
(**a**) XRD patterns of Ti_3_AlC_2_ and Ti_3_AlC_2_/Pd composites with different Pd contents; (**b**) XPS survey spectra of Ti_3_AlC_2_ and Ti_3_AlC_2_/Pd-3% composites, insert is the high-resolution XPS spectra of Pd 3d.

To confirm the composition of Ti_3_AlC_2_/Pd catalysts, XRD was performed, as shown in Figure 2a. The characteristic diffractions of Ti_3_AlC_2_ appear at 2θ = 9.4°, 33.9° 38.8°, 41.6°, and 48.2°, corresponding to the (002), (101), (104), (105), and (107) planes of Ti_3_AlC_2_, respectively [31]. For Ti_3_AlC_2_/Pd-1%, Ti_3_AlC_2_/Pd-3%, and Ti_3_AlC_2_/Pd-5% catalyst, the 2θ peaks at 40.2°, 46.7°, 67.9°, and 82.1° can be assigned to the (111), (200), (220), and (311) crystal planes of metallic Pd (JCPDS NO. 46-1043), respectively. These XRD results confirm that we have successfully synthesized Ti_3_AlC_2_/Pd composites catalysts. XPS characterization was further conducted to provide the chemical composition of prepared catalysts, and the corresponding result is shown in Figure 2b. As for the pure Ti_3_AlC_2_, all the peaks of Ti, C, Al, and O elements (in which the O element comes from the adsorbed oxygen species) can be detected in the survey XPS spectra. In contrast, in addition to the elements of Ti, C, Al, and O, the XPS peak of Pd species also appears for the Ti_3_AlC_2_/Pd-3% sample, further indicating that Pd is successfully loaded on the Ti_3_AlC_2_. Moreover, further observation by the high-resolution XPS spectra of Pd 3d (inset of Figure 2b) exhibits two peaks at 340.01 eV and 334.74 eV, which can be assigned to Pd 3d_3/2_ and Pd 3d_5/2_ of metallic Pd, respectively. The high-resolution XPS spectra of Ti 2p, C 1s, Al 2p, and Pd 3d are also provided, as shown in Figure S1. Figure S1a presents the Ti 2p XPS spectra of Ti_3_AlC_2_/Pd-1%, Ti_3_AlC_2_/Pd-3%, Ti_3_AlC_2_/Pd-5%, and Ti_3_AlC_2_/Pd. All the samples can be fitted with two categories of asymmetric Gaussian-Lorentzian curves representing the Ti 2p_3/2_ (~454.18 eV) and Ti 2p_1/2_ (~460.58 eV) spin-orbit of Ti-C component and Ti 2p_3/2_ (~458.64 eV) and Ti 2p_1/2_ (~464.47 eV) from TiO_2_ impurities [32,33]. Figure S1b presents the C 1s XPS spectra of Ti_3_AlC_2_/Pd-1%, Ti_3_AlC_2_/Pd-3%, Ti_3_AlC_2_/Pd-5%, and Ti_3_AlC_2_. The peaks at 284.78 eV are from C-based impurities, while the peaks at 281.28 eV and 288.83 eV are the Ti-C components and contamination of COO groups [34,35]. As for Al 2p XPS, both the metallic Al and the Al_2_O_3_ compounds are observed, as illustrated in Figure S1c. Specifically, the peaks at ~71.8 eV are the metallic Al components, while the peaks at ~74.1 eV are from Al_2_O_3_ impurities. Figure S1d shows the XPS Pd 3d spectra of as-prepared catalysts. Two asymmetric peaks at ~340.02 and ~334.74 eV binding energy are observed for Ti_3_AlC_2_/Pd-1%, Ti_3_AlC_2_/Pd-3%, and Ti_3_AlC_2_/Pd-5%, which correspond, respectively, to Pd^0^ 3d_3/2_ and Pd^0^ 3d_5/2_ [24,36]. The N_2_ adsorption/desorption isotherm curves of Ti_3_AlC_2_, Ti_3_AlC_2_/Pd-1%, Ti_3_AlC_2_/Pd-3%, and Ti_3_AlC_2_/Pd-5% exhibit typical type-IV isotherm with a H4 hysteresis loop (Figure S2a). According to the Brunauer–Emmett–Teller (BET) method, the BET specific surface area (S_BET_) is 1.3, 4.8, 2.6, and 5.7 m^2^ g^−1^ for Ti_3_AlC_2_, Ti_3_AlC_2_/Pd-1%, Ti_3_AlC_2_/Pd-3%, and Ti_3_AlC_2_/Pd-5%, respectively. These minor differences of S_BET_ might attribute to the local micro-nano structure differences of the samples or the instrument error. Figure S2b exhibits the pore size distributions of as-obtained samples derived from the Barrett–Joyner–Halenda (BJH) method. All the samples presented mostly 2–10 nm pores, which indicates that these samples feature both the microporous and mesoporous structures. To overview the textural characteristics, the summary of surface areas (S_BET_), pore volume (V_p_), and average pore diameter (D_p_) are given, as shown in Table S1. Moreover, the total Pd contents determined by inductively coupled plasma mass spectrometry (ICP-MS) are also obtained, as shown in Table S2. The actual loading of Pd for Ti_3_AlC_2_/Pd-1%, Ti_3_AlC_2_/Pd-3%, and Ti_3_AlC_2_/Pd-5% is 0.85 wt%, 2.80 wt%, and 4.88 wt%, which is almost consistent with the theoretical value of 1 wt%, 3 wt%, and 5 wt%, respectively.

**Figure 3:**
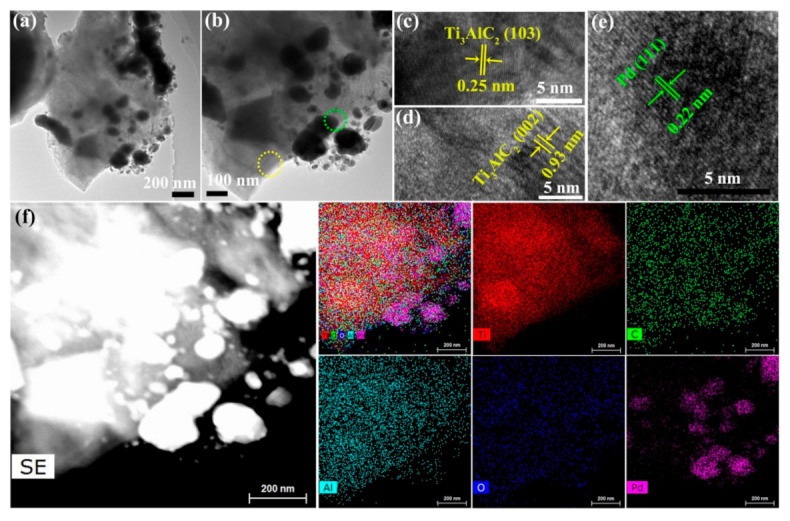
(**a**,**b**) TEM, (**c**–**e**) HRTEM and (**f**) EDX elemental mapping images of Ti_3_AlC_2_/Pd-3% catalyst. (The yellow circle in (**b**) focuses on Ti_3_AlC_2_ phase shown in (**c**,**d**), and the green circle focuses on Pd phase shown in (**e**)).

The morphology of Ti_3_AlC_2_, Ti_3_AlC_2_/Pd-1%, Ti_3_AlC_2_/Pd-3%, and Ti_3_AlC_2_/Pd-5% samples were investigated by SEM, as shown in Figure S3. It is important to note that the deposition of Pd here does not modify the Ti_3_AlC_2_ MAX phase structure in any way. As shown in Figure S3a,b, Ti_3_AlC_2_ MAX phase exhibits a typical three-dimensional and layered microstructure with irregular edges. The 3D nanostructured morphology possesses a large surface-to-volume ratio, which provides more reactive sites to participate in the catalytic process. After loading with Pd nanoparticle, the morphology of Ti_3_AlC_2_ is well retained, as shown in Figure S3c–h. Similar morphologies and structures, viz. thin sheets of Ti_3_AlC_2_, are observed for Ti_3_AlC_2_/Pd-1% (Figure S3c,d), Ti_3_AlC_2_/Pd-3% (Figure S3e,f), and Ti_3_AlC_2_/Pd-5% (Figure S3g,h). The structure and morphology of Ti_3_AlC_2_/Pd-3% samples are further investigated by TEM, as shown in Figure 3. As shown in Figure 3a,b, Ti_3_AlC_2_/Pd-3% exhibits predominantly accordion-like and multilayer morphology, which is typical morphology of bulk Ti_3_AlC_2_ MAX phase. Moreover, some fragments of Pd nanoparticles with an average particle size of 50–180 nm are evenly distributed and embedded in the Ti_3_AlC_2_ matrix. High-resolution TEM (HRTEM) characterization was further carried out to get more structural information on Ti_3_AlC_2_/Pd. Clear lattice fringes with a pitch of 0.25 nm and 0.93 nm are observed, which is ascribed to the (103) and (002) plane of Ti_3_AlC_2_, respectively (Figure 3c,d). On closer inspection of Figure 3e, the interplanar distance of the wrapped nanoparticles is about 0.22 nm, which corresponds to the (111) plane of metallic palladium. EDX elemental mapping of Ti, C, Al, O, and Pd elements over Ti_3_AlC_2_/Pd-3%, shown in Figure 3f, further validates the successful Pd load on the Ti_3_AlC_2_ support. The above results indicate that Pd nanoparticles have been successfully loaded on the Ti_3_AlC_2_ support.

**Figure 4:**
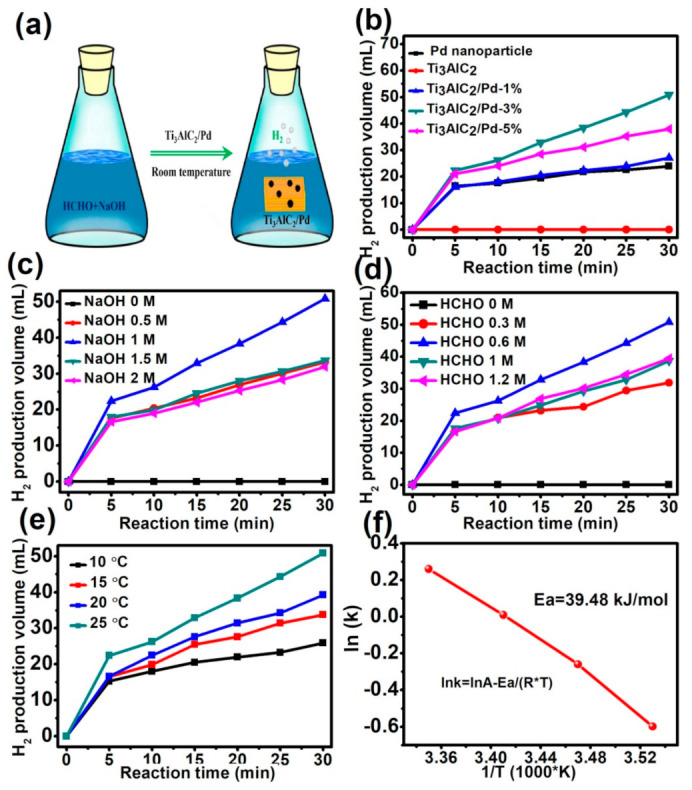
(**a**) Schematic illustration of the catalytic hydrogen production process over Ti_3_AlC_2_/Pd. (**b**) The comparison of hydrogen generation over different catalysts. Reaction condition: NaOH: 1 M, HCHO: 0.6 M, catalyst: 15 mg, reaction temperature: 25 °C. (**c**) Effect of NaOH concentrations on the hydrogen production. Ti_3_AlC_2_/Pd-3% catalyst: 15 mg, HCHO: 0.6 M, temperature: 25 °C; (**d**) effect of HCHO concentration on the hydrogen production. Ti_3_AlC_2_/Pd-3% catalyst: 15 mg, NaOH: 1 M, temperature: 25 °C. (**e**) Effect of reaction temperature on the hydrogen production, Ti_3_AlC_2_/Pd-3% catalyst: 15 mg, NaOH concentration: 1 M, HCHO concentration: 0.6 M. (**f**) Arrhenius plots for the H_2_ generation reaction over Ti_3_AlC_2_/Pd-3% catalyst.

In addition, the hydrogen production behavior of as-prepared Ti_3_AlC_2_/Pd was examined in alkaline formaldehyde solution at room temperature, and the reaction process scheme is illustrated in Figure 4a. As shown in Figure 4b, no hydrogen is detected for pure Ti_3_AlC_2_ support, indicating that Ti_3_AlC_2_ is only used as supported material rather than a reactive catalyst for catalyzing formaldehyde to produce hydrogen. By contrast, when Ti_3_AlC_2_/Pd catalysts are introduced into the reaction system, the hydrogen production is triggered immediately without any induction period (after adding the catalyst, the hydrogen was quickly overflowed, as can be clearly observed in the Video S1 in Supporting Information). Compared with Ti_3_AlC_2_/Pd-1% and Ti_3_AlC_2_/Pd-5% catalysts, Ti_3_AlC_2_/Pd-3% achieves the maximum hydrogen production after 30 min of reaction and is far higher than that of its Pd nanoparticle counterpart (typical TEM image and Pd size distribution histogram is shown in Figure S4a,b). After reaction for 30 min, 23.9, 30.1, 38.2, and 50.8 mL of hydrogen are produced over Pd nanoparticles, Ti_3_AlC_2_/Pd-1%, Ti_3_AlC_2_/Pd-5%, and Ti_3_AlC_2_/Pd-3%, respectively. The above results show that the greater the loaded mass of Pd supported on the Ti_3_AlC_2_/Pd does not mean a better hydrogen production performance. That is, only the catalyst with optimal Pd loading contents can exhibit the most excellent hydrogen production. On the one hand, excessive Pd nanoparticles might agglomerate heavily and lose active sites for hydrogen production, which hinders and reduces the utilization efficiency of surface Pd atoms. In addition, under our current experimental conditions, no carbon monoxide, carbon dioxide, or other gaseous products have been detected so far in all these catalytic processes.

To optimize the reaction conditions and obtain the maximum hydrogen production, the effects of NaOH concentration, HCHO concentration, and reaction temperature on the hydrogen production performance are systematically modulated and compared. As shown in Figure 4c, no hydrogen can be detected in the absence of NaOH. In contrast, quantitative hydrogen would be generated immediately when a certain concentration of NaOH is added to the reaction system. This indicates that alkaline conditions are indispensable for the catalytic hydrogen production process. In detail, as the NaOH concentration increases from 0.5 to 1.0 mol L^−1^, the hydrogen production during 30 min increases from 30.1 mL to 51.2 mL. However, further increased NaOH concentration to 1.5 mol L^−1^ and 2 mol L^−1^ leads to a gradually decreased hydrogen production of 33.5 and 31.7 mL, respectively. It was reported that when allowed to react with excess base, there was a competitive reaction of hydrogen production-Cannizzaro reaction (formaldehyde generates the Cannizzaro intermediate and subsequently forms methanol and formate) [20,37]. The Cannizzaro reaction may consume some of the formaldehyde molecules, blocking hydrogen production. Figure 4d shows the effect of HCHO concentration on the catalytic generation of H_2_ at 1 M NaOH. When the concentration of HCHO increases from 0.3 M to 0.6 M, the hydrogen production increases from 31.9 to 50.8 mL. However, the hydrogen production decreases upon increasing HCHO concentration to 1 M and 1.2 M. This can be well explained by the fact that when the concentration of formaldehyde is too high, the Cannizzaro reaction would occur simultaneously. Some of the formaldehyde molecules are transformed into methanol and formic acid. Therefore, these results clearly show that, to achieve a high hydrogen production rate, the concentration of HCHO and NaOH should be strictly controlled and at the optimal concentration.

The effect of the reaction temperature in the range of 10 °C to 25 °C on the rate of H_2_ production is also investigated, as shown in Figure 4e. It can be found that the hydrogen production rate increases gradually upon increasing reaction temperature. To be more specific, when the temperature increases from 10 °C to 25 °C, the hydrogen production after reaction of 30 min increases rapidly from 25.9 to 33.7, 39.2, and 50.8 mL, demonstrating that increasing the reaction temperature could promote the hydrogen production reaction. In addition, it should be noted that the initial H_2_ production rate after a reaction of 5 min reaches the highest. This result indicates that the rate of hydrogen production can be controlled by modulating the concentration of reactant formaldehyde. At the beginning of the reaction, the concentration of formaldehyde is the highest, and the reactions rate is the fastest. As the reaction time increases, the fewer reactants (that is, the lower concentration of formaldehyde), the lower the H_2_ production rate. More specifically, the rates of H_2_ generation at initial 5 min increase rapidly from 191.7 to 290.9 mL·min^−1^g^−1^ when the reaction temperature is increased from 10 to 25 °C, indicating that higher reaction temperature is in favor for the hydrogen production rection. Moreover, it shows that the amount of hydrogen produced is linearly dependent on the reaction time from 5–30 min at each reaction temperature, which means that such a hydrogen production reaction can be treated as a zero-order reaction. Thus, to fully understand the underlying principles of hydrogen production enhancement of Ti_3_AlC_2_/Pd-3% catalyst, the rate constant (lnk) is plotted as a function of reaction temperature (T), as is shown in Figure 4f. Accordingly, the reaction rate formula can be expressed as follows:

(1) $$k\;=\;\mathrm{Aexp}\left(-\mathrm{Ea}/\mathrm{RT}\right)$$

where k is the rate constant, A is the pre-exponential factor, Ea is the apparent activation energy, R is the gas constant, and T is the reaction temperature. According to the slope of Figure 3f, the activation energy of Ti_3_AlC_2_/Pd-3% catalyzed hydrogen production of formaldehyde is determined to be 39.48 kJ mol^−1^, which is considerably lower than 65 kJ mol^−1^ for hydrogen production without catalyst [38]. The results show that Ti_3_AlC_2_/Pd-3% catalyst reduces the activation energy of hydrogen production from alkaline solutions of formaldehyde. To sum up, the compound Ti_3_AlC_2_/Pd-3% catalyst can effectively catalyze formaldehyde aqueous solution to produce hydrogen at room temperature.

In order to verify the synergistic effect between the Pd nanoparticles and the Ti_3_AlC_2_ support on the hydrogen production performance from formaldehyde, we provide the morphology and hydrogen production performance of the uncalcined Ti_3_AlC_2_/Pd-3% sample (denoted as Ti_3_AlC_2_/Pd-3%-uc). As shown in Figure S5, Ti_3_AlC_2_/Pd-3%-uc exhibits a densely layer-stacked structure with micrometer size. As shown in Figure S6, only the characteristic XRD peaks of Ti_3_AlC_2_ phase can be found in Ti_3_AlC_2_/Pd-3%-uc, which may be due to its low Pd loading (3 wt%) and small Pd size and better Pd dispersion. Moreover, hydrogen production over Ti_3_AlC_2_/Pd-3%-uc is measured, as shown in Figure S7. The Ti_3_AlC_2_/Pd-3%-uc sample produces only 32.8 mL of hydrogen within 30 min, which is slightly higher than that of its pure Pd nanoparticle counterpart. In contrast, the calcined sample of Ti_3_AlC_2_/Pd-3% can generate 52.9 mL of hydrogen under the same reaction conditions, which is nearly 1.61 times that of the Ti_3_AlC_2_/Pd-3%-uc. Based on the results, we speculate that the calcination may improve the interaction between Pd and Ti_3_AlC_2_ support, and the strong metal-support interaction (SMSI) is possibly beneficial to boost mass transport processes at the interface and enhance the catalytic performance. Although the metal-support interaction is enhanced after calcination, compared to the uncalcined sample (TEM image of Figure S8 shows that the average size of Pd nanoparticles for Ti_3_AlC_2_/Pd-3%-uc is 20–30 nm), the Pd nanoparticles loaded on Ti_3_AlC_2_ support are obviously agglomerated and the particle size becomes larger, which is not conducive to the full utilization of the Pd active material. Therefore, further reducing the size of Pd nanoparticles, enhancing the metal-support interaction and ultimately improving the hydrogen production rate from alkaline formaldehyde solution are indispensable in the future research.

**Figure 5:**
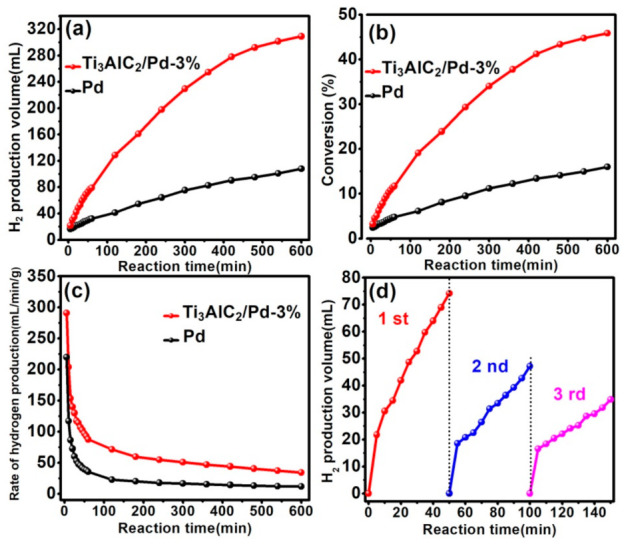
Comparison of the hydrogen generation over Ti_3_AlC_2_/Pd-3% and Pd nanoparticles: (**a**) Hydrogen production in 600 min. (**b**) Conversion rate of formaldehyde in 600 min. (**c**) Hydrogen production rate in 600 min. (**d**) Hydrogen production performance within three cycles. Reaction condition: catalyst: 15 mg, NaOH: 1 M, HCHO: 0.6 M, temperature: 25 °C.

To meet the needs of practical application, the catalyst with long-term stability and excellent recycling performance are highly expected. Thereby, the hydrogen production over Ti_3_AlC_2_/Pd-3% and Pd nanoparticles catalysts react in a prolonged 10 h is compared, as shown in Figure 5a. Generally, Ti_3_AlC_2_/Pd-3% catalyst possesses excellent hydrogen production performance with the reaction time extending, obtaining 318.9 mL of H_2_ after reaction for 10 h. In contrast, the yield of H_2_ over the Pd nanoparticles catalyst after 10 h is only 103.5 mL. Moreover, the HCHO conversion over Ti_3_AlC_2_/Pd-3% after reaction for 6 h is also established, as shown in Figure 5b. We observe that Ti_3_AlC_2_/Pd-3% catalyst shows higher HCHO conversion (47.8%) at room temperature (25 °C) compared to that of Pd nanoparticles catalyst (15.2%). The rate of hydrogen production over 6 h is also calculated, as shown in Figure 5c. The initial hydrogen production rate for Ti_3_AlC_2_/Pd-3% and Pd nanoparticle catalyst is 291.6 and 218.8 mL·min^−1^g^−1^. After reaction for 50 min, the hydrogen production rate for Ti_3_AlC_2_/Pd-3% is 103.7 mL·min^−1^g^−1^, which is 2.92 times that of its pure Pd nanoparticle counterpart (35.5 mL·min^−1^g^−1^). To check the recyclability of catalyst (Ti_3_AlC_2_/Pd-3%, herein, the 3 wt% of Pd content was not determined after the three reaction cycles), the solid catalyst is filtered off, washed thoroughly with deionized water and dried at ambient temperature. The Ti_3_AlC_2_/Pd-3% catalyst is reused up to evaluate the hydrogen production during three successive runs. Unfortunately, as exhibited in Figure 5d, the as-prepared Ti_3_AlC_2_/Pd-3% exhibits poor hydrogen production recyclability, which may be associated with weak metal-support interaction that promotes the leaching of active species during the catalysis reaction. Thus, the structural and morphological features of Ti_3_AlC_2_/Pd-3% after three cycles are examined. XRD patterns (Figure S9) of reused Ti_3_AlC_2_/Pd-3% exhibit the characteristic diffraction peaks of both Ti_3_AlC_2_ and Pd species. However, the morphological features of Ti_3_AlC_2_/Pd-3% have changed somewhat. As revealed by the TEM image in Figure S10, the palladium nanoparticles are no longer uniformly distributed on the Ti_3_AlC_2_/Pd-3%, and there is obvious palladium nanoparticles agglomeration. The bad recyclability of hydrogen production is thus most probably caused by the relatively weak metal-support interaction resulted from larger Pd nanoparticles and heavy agglomeration, which is often invoked to be critical in the catalytic reaction. As for the improvement in the chemoselectivity, the possible explanations of both theoretically and experimentally by other studies are as follows: (i) Pd transforms into a more stable adsorption structure through electronic effects, and the adsorbed molecules interact with the metal surface in a complex manner, which leads to changes in the H_2_ activation mechanism [39]. (ii) Through geometric effects, the in situ reduction of Pd with larger size and agglomeration decreases the number of adjacent adsorption sites [40]. Reducing the size of Pd nanoparticles (to prepare Pd single atoms or clusters) and enhancing the metal-support interaction (optimizing the preparation method of the catalyst, regulating the micro-nano structure of the support) are feasible approaches to address the weak cycling performance, which need to be strengthened for future work. However, more work and evidence should be given to better understand what is occurring as a function of the time of cycling in our system.

**Figure 6:**
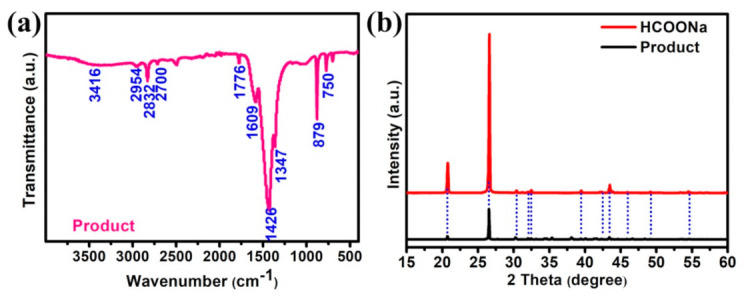
(**a**) FTIR spectra and (**b**) XRD pattern of the reaction products involved in catalytic HCHO oxidation over Ti_3_AlC_2_/Pd-3% catalyst. NaOH: 1 M, HCHO: 0.6 M, catalyst: 15 mg, reaction temperature: 25 °C.

**Scheme 1:**
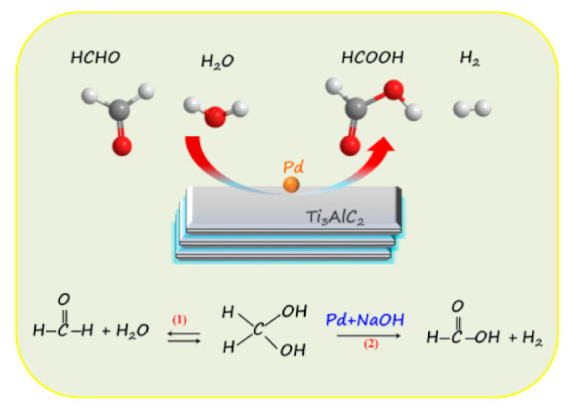
Schematic illustration of the catalytic formaldehyde to produce hydrogen over Ti_3_AlC_2_/Pd catalyst.

Except for the hydrogen detected in the gas phase, the intermediates that existed in the aqueous solution after formaldehyde oxidation catalyzed by Ti_3_AlC_2_/Pd-3% have also been evaluated using fourier transform infrared spectroscopy (FTIR) to investigate the reaction mechanism. As shown in Figure 6a, three bands at 2700, 2832, and 2954 cm^−1^ are typically characteristic of formate species, corresponding to the C-H stretching vibration, ν(C-H), symmetric and asymmetric stretching vibrational modes of the OCO moiety (i.e., ν_s_(OCO) and ν_as_(OCO), respectively) [41]. The bands at 1609 cm^−1^ and 1347 cm^−1^ are characteristic modes of monodentate species, which corresponding to the ν_as_(OCO) and ν_s_(OCO) [42]. Molecularly adsorbed HCHO molecules have also been detected, with characteristic IR bands of the ν(CO) mode at 1776 cm^−1^ [43,44]. Moreover, the O-H stretching vibration ν(OH) in formate species with the wide and strong bands is observed at 1426 cm^−1^. The two bands at 879 and 750 cm^−1^ are characteristics of C-O bending vibration (δ(C-O)). Based on the FTIR results, it is concluded that the additional main products in the aqueous solution of formaldehyde catalyzed by Ti_3_AlC_2_/Pd-3% are formate, that is, sodium formate. In addition, the obtained products are further analyzed by XRD (Figure 6b), and the main diffraction peaks can be well assigned to and matched with pure HCOONa, which is well in accordance with the FTIR results and reported literature [22]. Based on these results, we propose a possible reaction pathway for the formation of H_2_ from catalyzing oxidation of formaldehyde, as shown in Scheme 1. In this reaction system preceded at room temperature and at low concentrations of formaldehyde, one molar of formaldehyde and one molar H_2_O is catalyzed by the Ti_3_AlC_2_/Pd to generate one molar of HCOOH and one molar of H_2_ in alkaline solution. More specifically, formaldehyde in water can be almost completely hydrated to form methylene glycol (in Equation (1) of Scheme 1) [20,37]. Subsequently, under alkaline conditions, methylene glycol catalyzed by Pd nanoparticles produces hydrogen and formic acid. However, the source of the hydrogen atoms of H_2_ produced by this reaction is not yet fully understood. Early results suggested that one hydrogen atom originates from formaldehyde and the other from water molecular in the formation of H_2_ [20]. For the by-products of formic acid and sodium formate produced in this experiment, no strategies have been proposed so far to deal with them. The removal of formic acid or sodium formate produced in this experiment in an efficient and inexpensive way is still an open topic that needs to be strengthened for future work.

## 4. Conclusions

In conclusion, palladium nanoparticles supported on Ti_3_AlC_2_ catalysts have been prepared, and they exhibited an excellent catalytic activity for the oxidation of formaldehyde and the production of hydrogen under room temperature and atmospheric pressure. After reaction for 600 min, the Ti_3_AlC_2_/Pd catalyst with Pd loading of 3 wt% exhibited the highest hydrogen production (318.9 mL), and far exceeded that of conventional Pd nanoparticles (103.5 mL). Moreover, the effect of NaOH concentration, formaldehyde concentrations, reaction temperature, and Pd loading on the production of hydrogen over as-prepared Ti_3_AlC_2/_Pd have also been analyzed systematically to optimize the reaction conditions. The optimized experimental conditions for hydrogen production are: NaOH: 1 M, HCHO: 0.6 M, Pd loading: 3 wt%. The calculated 39.48 kJ mol^−1^ is much lower than that of the reported one (65 kJ mol^−1^). The prepared Ti_3_AlC_2_/Pd catalysts, as well as the catalytic process, could act as a “two birds with one stone” effect, that is, they not only eliminate noxious formaldehyde but also generate clean hydrogen.

## Data Availability

Not applicable.

## Supplementary Materials

The following supporting information can be downloaded at: https://www.mdpi.com/article/10.3390/nano12050843/s1. Figure S1: XPS results; Figure S2: N_2_ adsorption/desorption isotherms and pore size distributions; Table S1: surface areas (S_BET_), pore volume (Vp) and average pore diameter (Dp); Table S2: ICP-MS results; Figure S3: SEM images; Figure S4: TEM images; Figure S5: SEM images; Figure S6: XRD patterns; Figure S7: H_2_ production versus reation time; Figure S8: HRTEM image; Figure S9: XRD patterns; Figure S10: TEM images; Video S1: hydrogen bubbling.
