# Supplementary material for: Ti3AlC2/Pd Composites for Efficient Hydrogen Production from Alkaline Formaldehyde Solutions
Source: Nanomaterials (Basel). 2022 Mar 2;12(5):843. doi: 10.3390/nano12050843 (PMC8912652; doi:10.3390/nano12050843)
Supplement: Supplementary file 1 [file nanomaterials-12-00843-s001.zip › nanomaterials-1617754-SI-Additional figures and tables.pdf]

Supporting Information

Ti_3AlC_2/Pd Composites for Efficient Hydrogen Production from Alkaline Formaldehyde Solutions

Xiaogang Liu *, Wenjie Chen and Xin Zhang

College of Chemistry and Chemical Engineering, Xinyang Normal University, Xinyang 464000, China; chen2wen9jie5@163.com (W.C.); zhangxin109zz@163.com (X.Z.)

* Correspondence: lxg133298@163.com

Additional figures and tables

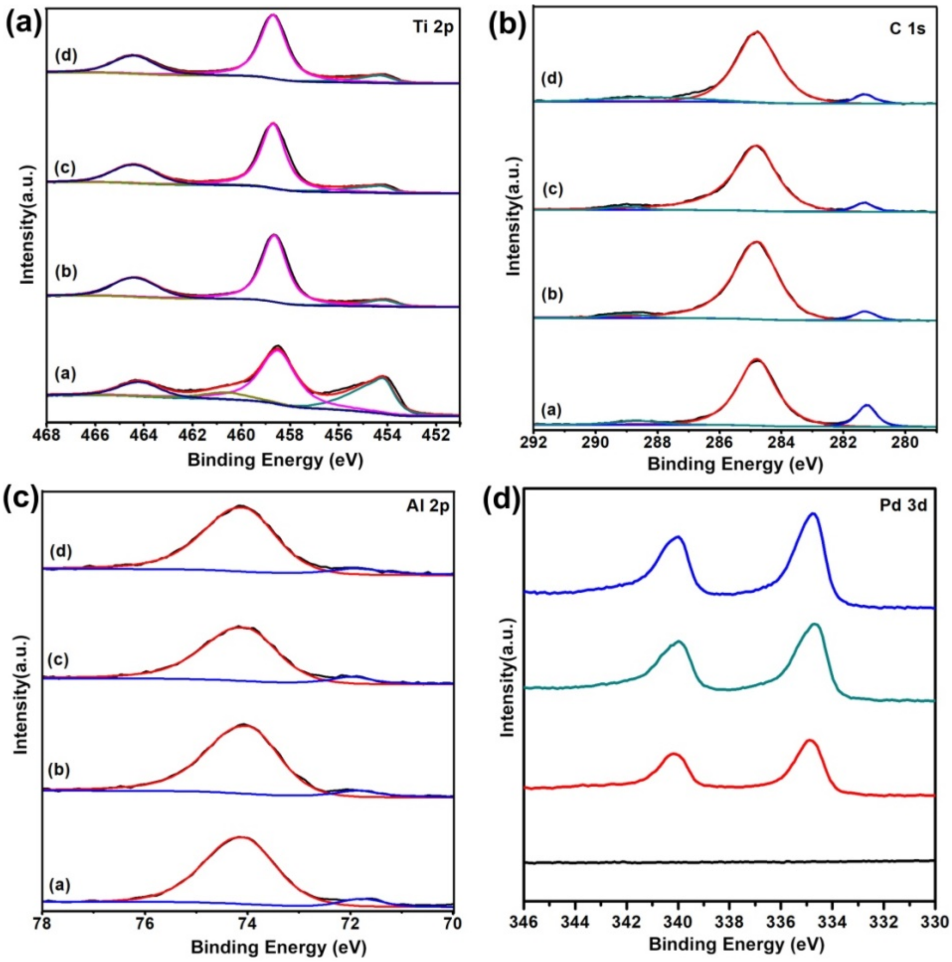

Figure S1. (a) C 1s high-resolution spectrum of Ti_3AlC_2 and Ti_3AlC_2/Pd composites with different Pd contents. (b) Ti 2p high-resolution spectrum of Ti_3AlC_2 and

Ti_3AlC_2/Pd composites with different Pd contents. (c) Al 2p high-resolution spectrum of Ti_3AlC_2 and Ti_3AlC_2/Pd composites with different Pd contents. (d) Pd 3d high-resolution spectrum of Ti_3AlC_2 and Ti_3AlC_2/Pd composites with different Pd contents. XPS curves marked as (a), (b), (c), (d) represent the samples of Ti_3AlC_2, Ti_3AlC_2/Pd-1%, Ti_3AlC_2/Pd-3%, Ti_3AlC_2/Pd-5%, respectively.

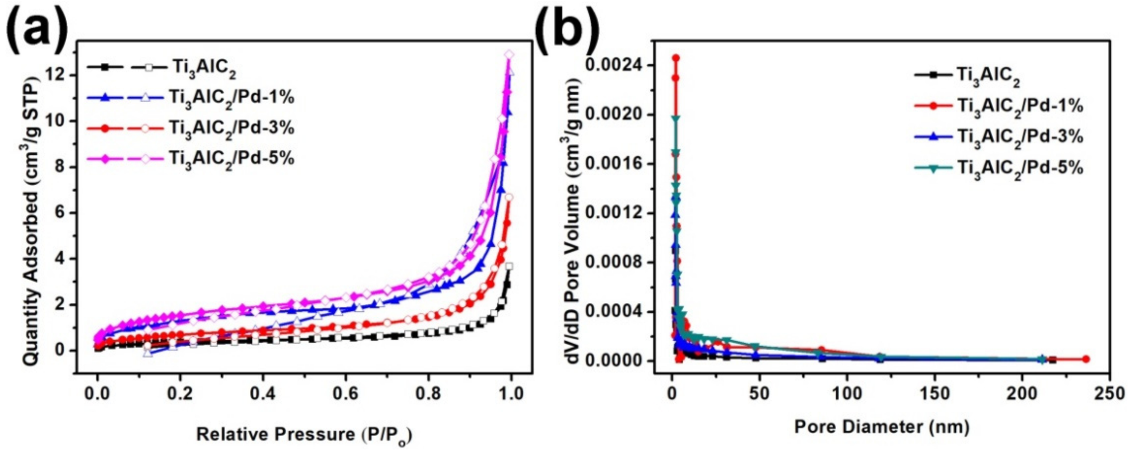

Figure S2. (a) N_2 adsorption/desorption isotherms for Ti_3AlC_2 and Ti_3AlC_2/Pd composites. (b) The corresponding pore size distributions.

Table S1. Summary of surface areas (S_{BET}), pore volume (V_p) and average pore diameter (D_p) of Ti_3AlC_2, Ti_3AlC_2/Pd-1%, Ti_3AlC_2/Pd-3% and Ti_3AlC_2/Pd-5%.

Catalyst	S_{BET} (m^2/g)	V_p (cm^3/g)	D_p (nm)
Ti_3AlC_2	1.3524	0.002146	10.5674
Ti_3AlC_2/Pd-1%	4.8802	0.007198	10.7277
Ti_3AlC_2/Pd-3%	2.6375	0.004485	10.9468
Ti_3AlC_2/Pd-5%	5.7121	0.009405	11.3767

Table S2. ICP-MS analysis of as-prepared Ti_3AlC_2/Pd-1%, Ti_3AlC_2/Pd-3%, and Ti_3AlC_2/Pd-5% samples.

Samples	Pd contents (wt. %)
Ti_3AlC_2/Pd-1%	0.85
Ti_3AlC_2/Pd-3%	2.80
Ti_3AlC_2/Pd-5%	4.88

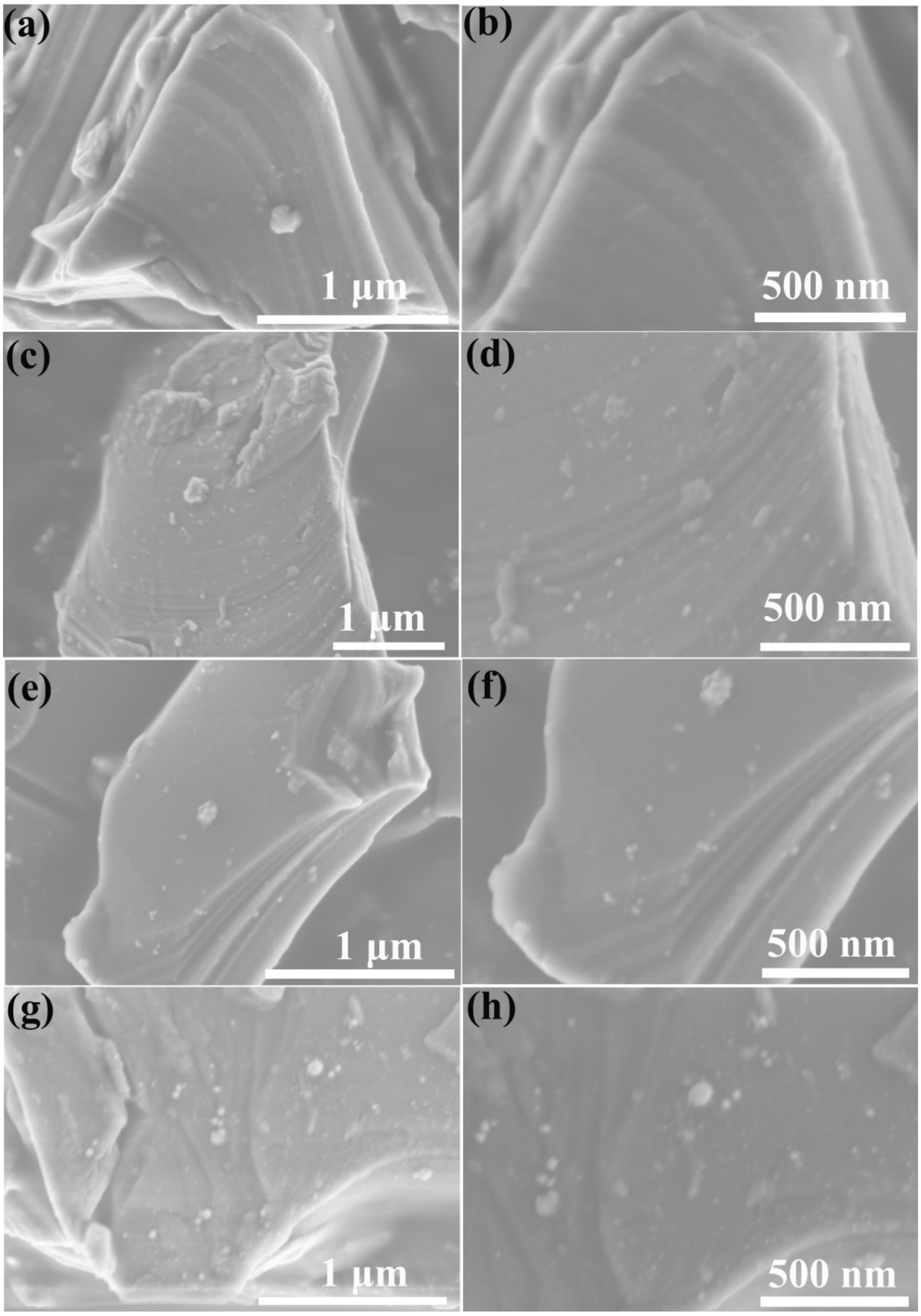

Figure S3. SEM images of (a,b) Ti_3AlC_2, (c,d) Ti_3AlC_2/Pd-1%, (e,f) Ti_3AlC_2/Pd-3%, (g,h) Ti_3AlC_2/Pd-5% catalysts.

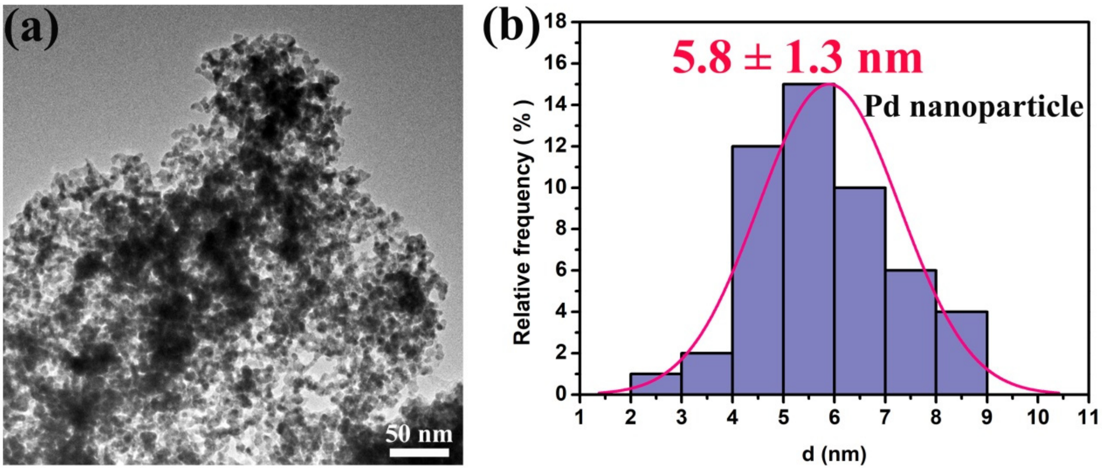

Figure S4. (a) TEM images of Pd nanoparticles prepared by $NaBH_4$ reduction. (b) histogram of Pd particle size. Pd nanoparticles were prepared by $NaBH_4$ reduction method. In detail, 30 mL of deionized water was mixed with 1 wt % of H_2PdCl_4 solution. Then 10 mL of 0.5 M $NaBH_4$ (freshly prepared) was added drop-wise into the solution, the color of the solution changed from white to gray through continuous magnetic stirring. The reaction solution was stirred continuously for 24 h to complete the reduction reaction. The Pd nanoparticles catalysts were collected by centrifugation, washed four times with deionized water and dried for 8 h in an oven at 60 °C.

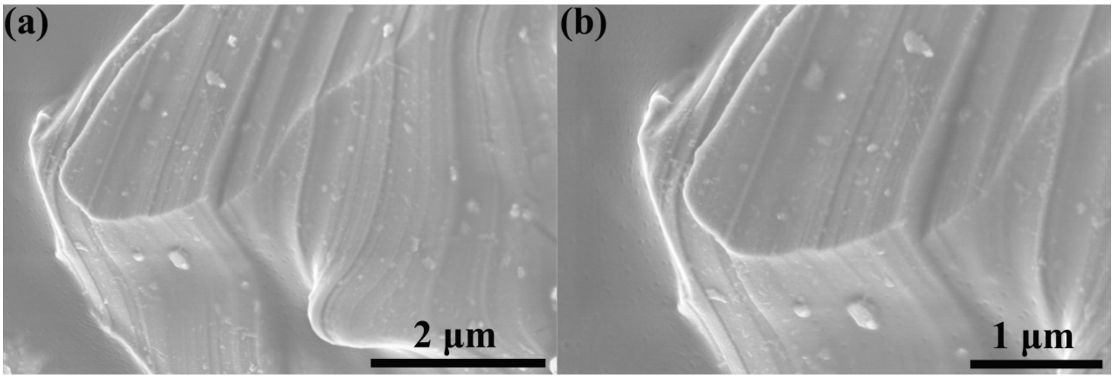

Figure S5. (a,b) SEM images of Ti_3AlC_2/Pd-3%-uc.

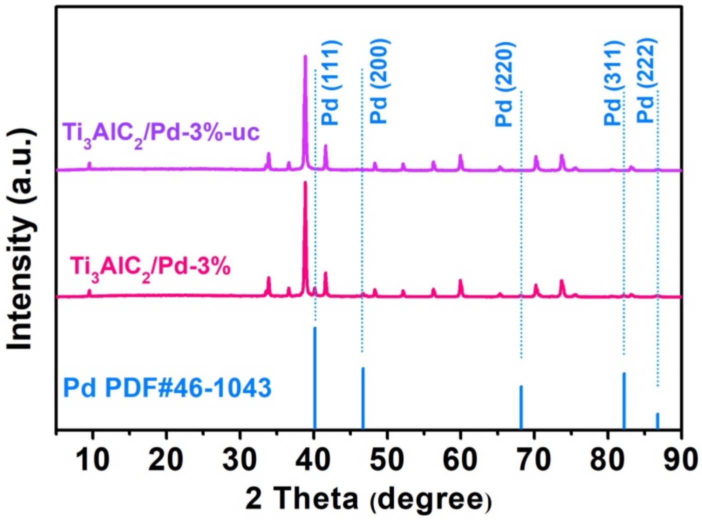

Figure S6. XRD patterns of Ti_3AlC_2/Pd-3% and Ti_3AlC_2/Pd-3%-uc composites.

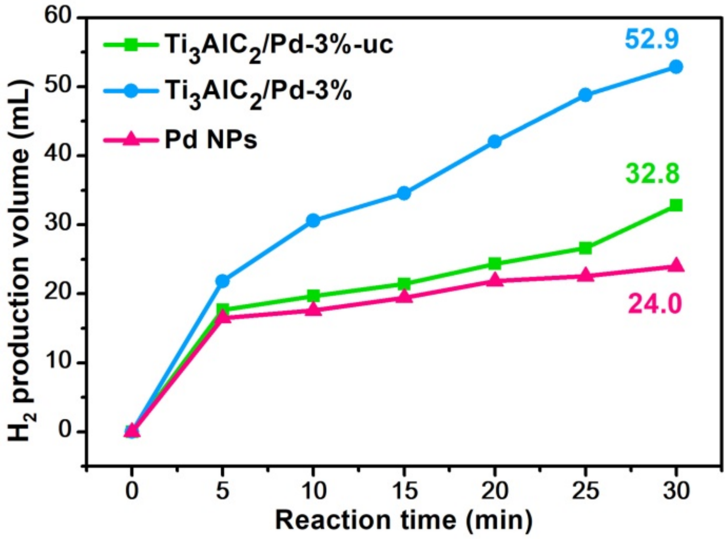

Figure S7. Hydrogen production of Ti_3AlC_2/Pd-3%, Ti_3AlC_2/Pd-3%-uc and Pd nanoparticles. Reaction condition: catalyst: 15 mg, NaOH: 1 M, HCHO: 0.6 M, temperature: 25 °C.

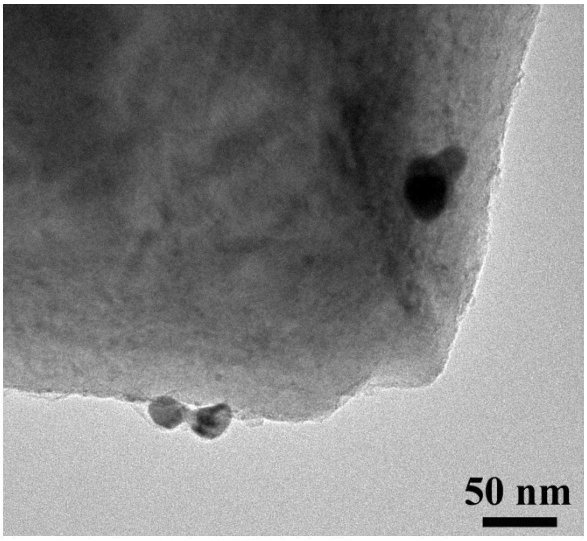

Figure S8. HRTEM image of Ti_3AlC_2/Pd-3% -uc.

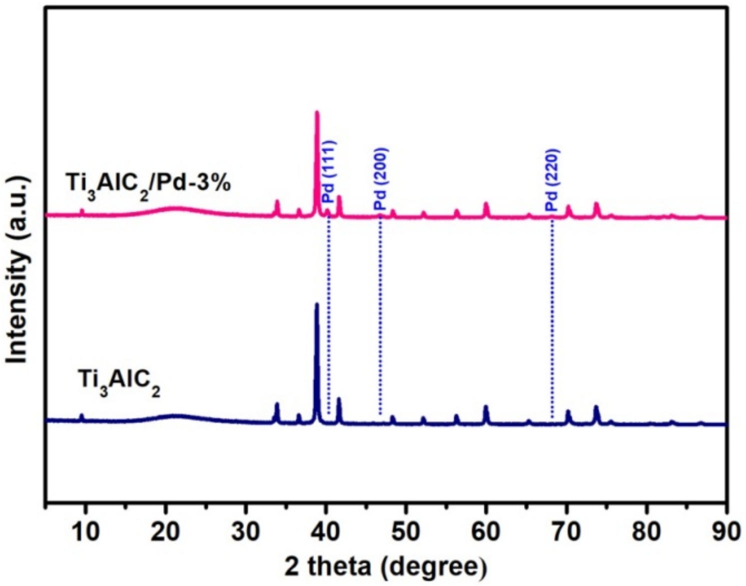

Figure S9. XRD patterns of Ti_3AlC_2/Pd-3% composites after catalytic reaction.

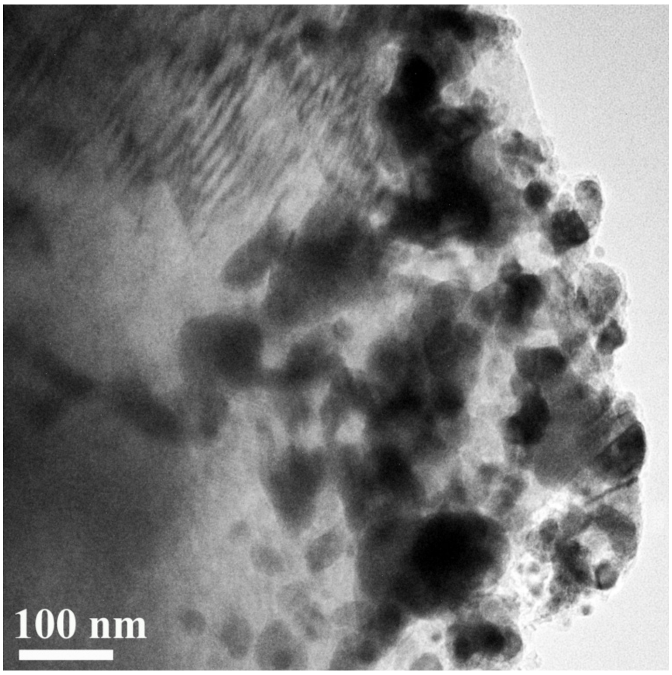

Figure S10. TEM images of Ti_3AlC_2/Pd-3% catalyst after three catalytic cycles.
